# Jab1 is a target of EGFR signaling in ERα-negative breast cancer

**DOI:** 10.1186/bcr2105

**Published:** 2008-06-06

**Authors:** Jiaxu Wang, Rebecca O Barnes, Nathan R West, Melanie Olson, Jenny E Chu, Peter H Watson

**Affiliations:** 1 Deeley Research Center, BC Cancer Agency, Vancouver Island Center, 2410 Lee Avenue, Victoria, BC, V8R 6V5, Canada; 2 University of Victoria, PO Box 1700 STN CSC, Victoria, BC, V8W 2Y2, Canada; 3 Molecular Oncology and Breast Cancer Program, BC Cancer Agency, 600 West 10th Avenue, Vancouver, BC, V5Z 4E6, Canada; 4 Department of Pathology, BC Cancer Agency, 600 West 10th Avenue, Vancouver, BC, V5Z 4E6, Canada

## Abstract

**Introduction:**

c-Jun activation domain-binding protein-1 (Jab1) is a multifunctional signaling protein that previously has been shown to be a master regulator of a poor prognostic gene signature in invasive breast cancer and to mediate the action of S100A7. Since epidermal growth factor receptor (EGFR), like S100A7, is often expressed in estrogen receptor-alpha-negative (ERα^-^) breast cancer, we set out to investigate the role of Jab1 in mediating EGFR signaling, another facet of the ERα^- ^phenotype.

**Methods:**

MDA-MB-231 and MDA-MB-468 ERα^-^/EGFR^+ ^cell lines were assessed for localization of Jab1 and levels of downstream genes by immunofluorescence and nuclear protein extract assay following treatment with epidermal growth factor (EGF) and extracellular signal-regulated kinase (ERK) pathway inhibitor. A cohort of 424 human breast tumors was also assessed by immunohistochemistry.

**Results:**

EGF treatment of cell lines resulted in increased Jab1 nuclear expression. This effect was inhibited by the ERK pathway inhibitor, PD98059. EGF treatment was also associated with colocalization of pERK (phosphorylated ERK) and Jab1 as well as regulation of the Jab1 downstream target gene, p27. When Jab1 activity was knocked down, p27 levels were restored to pre-EGF treatment level. Analysis of EGFR and Jab1 expression in a cohort of invasive breast tumors by tissue microarray and immunohistochemistry confirmed a relationship between EGFR and increased nuclear Jab1 within the ERα^- ^subset (n = 154, *P *= 0.019). The same association was also confirmed for S100A7 and Jab1 (*P *= 0.036), and high Jab1 nuclear expression was most frequent in tumors that were positive for both EGFR and S100A7 (*P *= 0.004).

**Conclusion:**

Jab1 is a target of EGFR signaling in ERα^- ^cell lines and breast tumors and therefore may be a common central factor and potential therapeutic target for important cell signaling pathways in ERα^- ^breast cancer.

## Introduction

Recent therapeutic advances have improved survival for many patients with breast cancer. These advances have been most impressive for targeted therapies, such as those targeting the estrogen receptor (ER) and the human epidermal growth factor receptor (EGFR) 2 (Her2). These advances have specifically benefited the subsets of patients with tumors that exhibit ERα^+ ^or Her2^+ ^phenotypes, respectively. Other subsets of tumors such as the so-called 'triple-negative' breast tumors, ERα^-^/progesterone receptor-negative (PR^-^)/Her2^-^, remain difficult to treat. The ERα^- ^phenotype, which includes the triple-negative phenotype, has dominated clinical and biological consideration of breast cancer for many years and has been reproducibly shown in microarray studies to be distinct from ERα^+ ^breast cancer [1,2]. Identification of key signaling molecules and pathways relevant to ERα^- ^breast cancer is therefore an important step toward the goal of improving breast cancer therapy [3-5].

We and others have previously identified genes that are highly associated with the ERα^- ^phenotype, including EGFR and S100A7 [6-11]. Epidermal growth factors (EGFs) are important in the biology of both normal and malignant breast tissue, exerting their effects through their tyrosine kinase growth factor receptors. EGFR expression is strongly associated with the ERα^- ^phenotype [12-14] such that there is a strong inverse relationship between EGFR and the steroid receptor, ERα [11,15]. S100A7 (psoriasin) is a small calcium-binding protein belonging to the S100 gene family [16,17]. It is highly expressed in some ductal carcinoma *in situ *(DCIS) [18-20] and invasive breast [18] carcinomas. Within both of these stages, S100A7 expression is strongly related to the ERα^- ^phenotype [6,8].

c-Jun activation domain-binding protein-1 (Jab1) is a multifunctional signaling protein and is a target of S100A7 that can mediate many of its biological effects, including induction of nuclear factor-kappa B (NF-κB) and promotion of cell survival [21,22]. Additional evidence that Jab1 is a key gene in breast cancer progression comes from the recent finding that it is a downstream target for Her2 [23]. Furthermore, Jab1 has been found to interact with c-myc to act as a master regulator of the 'wound response' gene signature in breast cells [24,25]. The wound response signature represents a conserved cluster of gene responses to changes in serum, exclusive of known proliferation response genes. It can be generated in epithelial and fibroblast cells and is associated with poor outcome in invasive breast cancer. Jab1 also interacts with many components of known cell signaling pathways in the context of both phosphorylation and proteasomal activities, typically resulting in translocation of Jab1 to the nucleus and modification of activity in downstream pathways. These interactions result in increased activation protein-1 (AP-1) [26] and NF-κB [22] activity and degradation of the cell cycle inhibitor p27 (Kip1) [27] and the transforming growth factor-β signaling component Smad4 [28].

Taken together, these findings implicate Jab1 as an important factor in several signaling pathways in breast cancer. Since the S100A7 gene is strongly associated with the ERα^- ^phenotype and our studies have implicated Jab1 as a mediator of S100A7 action [7,22], we set out to examine the possibility that Jab1 may be an important component of the mechanism of action of other key ERα^-^-associated genes, focusing here specifically on EGFR.

## Materials and methods

### Cell lines, antibodies, and reagents

Human breast carcinoma cell lines MDA-MB-468 and MDA-MB-231 (both ERα^- ^and EGFR^+ ^and derived from invasive breast carcinomas) were cultured in Dulbecco's modified Eagle's medium supplemented with 10% fetal bovine serum under standard conditions as previously described [8]. The antibodies used for immunoblotting and immunoprecipitation were Jab1 (1:3,000) (Sigma-Aldrich, Oakville, ON, Canada); p27 (1:200) (BD Biosciences, Mississauga, ON, Canada); Lamin A/C (1:1,000), pEGFR (phosphorylated EGFR) (1:1,000), extracellular signal-regulated kinase (ERK) (1:1,000), phosphorylated ERK (pERK) (1:1,000), AKT (1:2,000), and pAKT (1:1,000) (Cell Signaling Technology, Inc., Danvers, MA, USA); EGFR (1:1,000) (VWR International, Mississauga, ON, Canada); and glyceraldehyde 3-phosphate dehydrogenase (GAPDH) (1:4,000; Advanced ImmunoChemical Inc., Long Beach, CA, USA). The antibody to S100A7 was a rabbit polyclonal generated and described previously [6,21]. Goat anti-mouse and goat anti-rabbit IgG secondary antibodies were purchased from Santa Cruz Biotechnology, Inc. (Santa Cruz, CA, USA). All EGF treatments were for 4 hours and, with the exception of the EGF dose experiments, were 50 ng/mL (Millipore Corporation, Billerica, MA, USA). Treatments with ERK inhibitor PD98059 were at 20 μM for 4 hours (Cell Signaling Technology, Inc.).

### Immunofluorescence, nuclear extraction, and immunoblotting

Following treatment with selected reagent (EGF or PD98059), cells were fixed with 3.7% formaldehyde, permeabilized with 0.1% Triton X-100, and blocked with 0.2% bovine serum albumin. Cells then were stained for Jab1 (1:50) using the primary antibodies described above and Alexa Fluor 488-conjugated goat anti-rabbit IgG secondary antibody (1:100) (Invitrogen Corporation, Carlsbad, CA, USA). For double-immunostaining of Jab1 and pERK or p27, cells first were stained for Jab1 as described above and then were stained for pERK (1:100) or p27 (1:100) using the primary antibodies described above and Alexa Fluor 594-conjugated chicken anti-mouse IgG secondary antibody (1:100) (Invitrogen Corporation). Immunofluorescence images were captured using a Leica DM 6000B immunofluorescence microscope (Leica, Wetzlar, Germany), and image analysis was performed using OpenLab 4.0.4 software (Improvision Ltd., Coventry, UK).

Nuclear extracts were prepared by rinsing culture dishes with phosphate-buffered saline (PBS) and then resuspending cells in a nuclear extracting lysis buffer (10 mM HEPES [4-(2-hydroxyethyl)-1-piperazineethanesulfonic acid] [pH 7.9], 10 mM KCl, 0.1 mM EDTA [ethylene diamine tetraacetic acid], and 0.1 mM EGTA [ethylene glycol tetraacetic acid]) and incubating for 20 minutes on ice. Cells were further fractionated by adding 25 μL of Nonidet P-40 (10%), vortexing for 10 seconds, and centrifuging at 15,000 *g *for 10 minutes at 4°C. The pellet (nuclei) was then resuspended in 50 mM HEPES (pH 7.3), 150 mM NaCl, 2.5 mM EGTA, 10% glycerol, 0.1% Tween-20, 1 mM NaF, 1 mM DTT (dithiothreitol), 0.1 mM Na_3_VO_3_, and one tablet of EDTA-free protease inhibitor (Roche Diagnostics, Basel, Switzerland) per 10 mL, incubated 20 minutes on ice, and then boiled prior to loading.

Protein samples were separated by SDS-PAGE (4% to 12% acrylamide) and transferred to 0.2 μm nitrocellulose (Bio-Rad Laboratories, Inc., Hercules, CA, USA). After blocking in 5% skim milk powder in PBST (PBS + 0.05% Tween-20) for 30 minutes, blots were rinsed in PBST and then incubated with the primary antibody overnight in PBST at 4°C. Blots were washed in PBST for 10 minutes, three times, and then were incubated with the appropriate secondary antibody for 1 hour, followed by washing in PBST for 10 minutes, three times. Blots were developed by chemiluminescence (enhanced chemiluminescence; made in house) and were exposed to X-OMAT Kodak film (Eastman Kodak Company, Rochester, NY, USA). For all assays, at least three separate experiments were performed.

### Knockdown of Jab1

Jab1 expression was inhibited by transfecting cells with a pool of four different Jab1-specific short interfering RNA (siRNA) duplexes (Dharmacon, Inc., Chicago, IL, USA). Scrambled siRNA was used as a non-targeting control (Dharmacon, Inc.). siRNA transfection was carried out using DharmaFECT 1 transfection reagent/vehicle (Dharmacon, Inc.) according to manufacturer recommendations. siRNA was transfected at a concentration of 100 nM, after which cells were cultured for 48 hours prior to lysis and protein harvest. Densitometry of Western blots was conducted using Adobe Photoshop (Adobe Systems Incorporated, San Jose, CA, USA). Densitometry results for p27 were normalized to GAPDH within each treatment. Statistical analysis of p27 densitometry was performed with JMP software (version 7.0) (SAS Institute Inc., Cary, NC, USA) using *t *tests.

### Tissue microarray breast cancer cohort

After the institutional research ethics board gave ethical approval, a tissue microarray (TMA) was obtained from the Manitoba Breast Tumor Bank (Winnipeg, MB, Canada) to investigate the relationship between Jab1 and EGFR and S100A7 in breast tumors *in vivo*. The TMA was constructed from duplicate 0.6-mm tissue cores that were removed from the central portion of a representative paraffin block from each tumor and arrayed within one of seven paraffin blocks, using a tissue arrayer (Beecher Instruments, Inc., Sun Prairie, WI, USA). The TMA included interpretable cores from 424 invasive breast carcinomas. Case selection was designed to mirror the distribution of major prognostic clinical-pathological features (size, grade, and lymph node and ER status) that the entire tumor bank collection accrued over the period 1992 to 2002 and was also based on the following criteria: (a) a minimum patient follow-up of 60 months and tumors that had (b) an invasive component of greater than 20% of the tissue section and (c) less than or equal to 10% of the normal epithelial content. ER^- ^status was defined by ligand-binding assay (LBA) criteria of less than 3 fmol/mg protein. The criteria for interpretation of the variables were as follows: (a) PR^+ ^status was defined as greater than or equal to 15 fmol/mg protein by LBA, (b) tumor grading was according to the Nottingham system, and (c) tumor size was classified as either small (≤ 20 mm) or large (>20 mm). Patients received a range of treatments, including local radiotherapy (n = 163) and systemic hormonal and/or chemotherapy (n = 375). Patient outcome was defined as the time from initial surgery to the date of death attributable to breast cancer only.

### Immunohistochemistry and statistical analysis

Immunohistochemistry (IHC) staining for Jab1, EGFR, and S100A7 was performed using an automated tissue immunostainer (Ventana Medical Systems, Inc., Tucson, AZ, USA) and using bulk reagents supplied by the manufacturer (IHC protocol previously described [6,21]). Primary antibody incubation for Jab1 and S100A7 was 32 minutes. Tumor cell staining was scored for each protein in semi-serial sections by a single observer (PHW) but in independent sessions for each protein to ensure blinded independent scoring. For Jab1 and S100A7, only nuclear expression was scored as cytoplasmic signals were generally weak and difficult to quantify. IHC staining was scored using a semi-quantitative IHC score (IHC score = [percentage of positive neoplastic epithelial cells] × [staining intensity ranked from 0 to 3]) that ranged from 0 to 300. In univariate analysis, cut-points for Jab1 and S100A7 were those used in previous studies [6,29] to distinguish low from high expression (Jab1 IHC scores of greater than 50, corresponding to nuclear expression in greater than 50% of tumor cells, and S100A7 IHC scores of greater than 0) or EGFR IHC scores of greater than 100, corresponding to 2+ or 3+ intensity as used for the clinical assessment of Her2 [30]. Statistical analysis was performed with JMP software (version 7.0) (SAS Institute Inc.) and GraphPad Prism (version 4.2) (GraphPad Software, Inc., San Diego, CA, USA) using Spearman correlation, chi-square, Mann-Whitney *t *test, or log-rank test as appropriate.

## Results

### Treatment with EGF influences localization of Jab1

**Figure 1:**
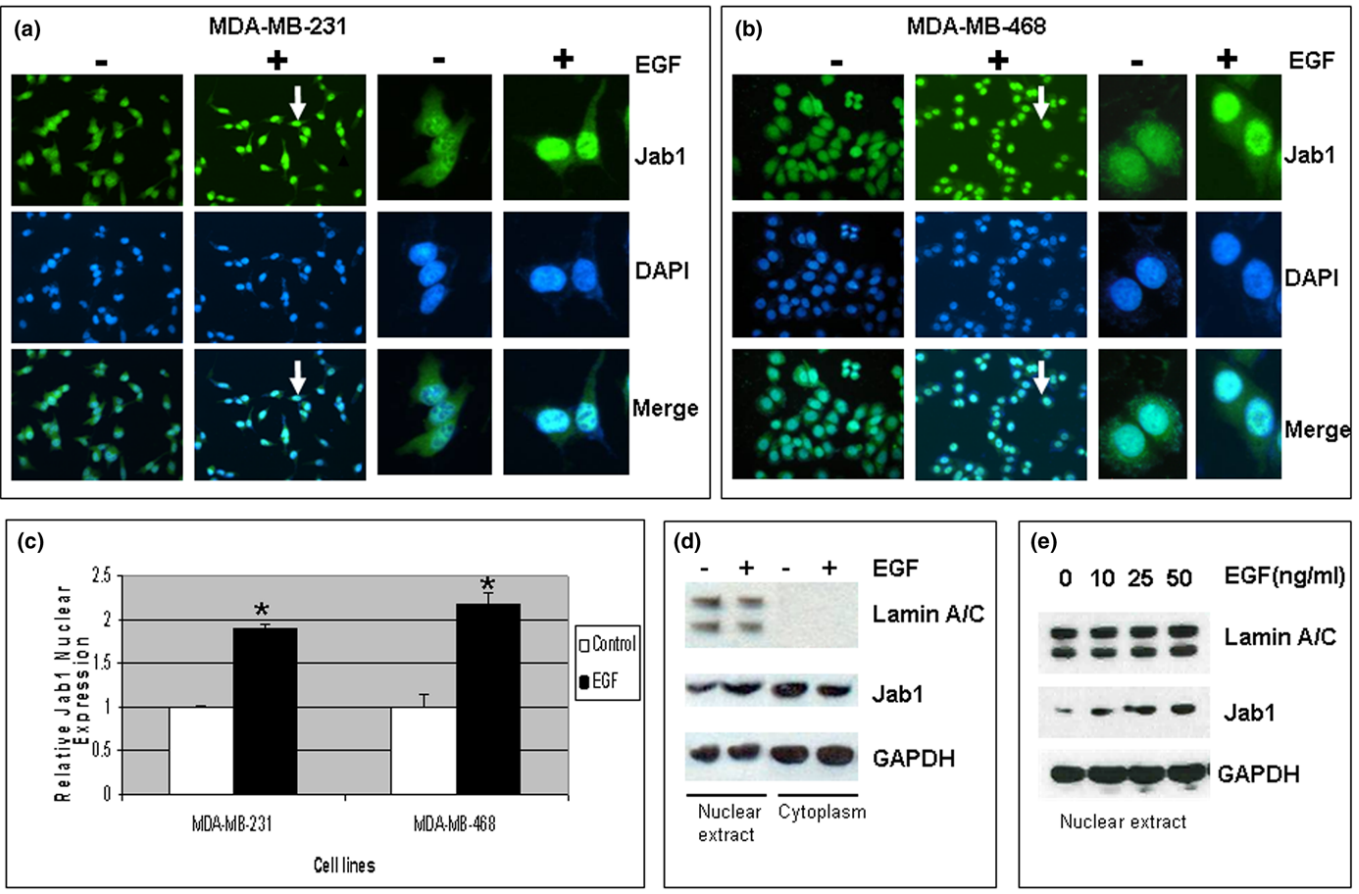
Epidermal growth factor (EGF) stimulates increased nuclear localization of Jab1 in MDA-MB-231 and MDA-MB-468 breast cancer cell lines. **(a, b) **Immunofluorescence assays. **(c) **Quantitative image analysis of nuclear Jab1 (bars represent mean ± standard deviation). Statistical analysis was by *t *test (*P < 0.01). **(d) **Increased expression of Jab1 in the nucleus relative to cytoplasm, detected by Western blot in MDA-MB-231 nuclear extracts. Lamin A/C was included as a positive control for the nuclear extract, and GAPDH was included as a loading control. **(e) **Increased Jab1 nuclear expression positively correlated to EGF dose. DAPI, 4'-6-diamidino-2-phenylindole; GAPDH, glyceraldehyde 3-phosphate dehydrogenase; Jab1, c-Jun activation domain-binding protein-1.

Jab1 has been shown previously to exist in both the nucleus and cytoplasm of different cell types. However, it has been shown that interactions between Jab1 and many of its downstream targets are associated with translocation of Jab1 to the nucleus. These include interaction with AP-1 [31], NF-κB [32], and p27 [32]. To determine whether Jab1 translocation is affected by EGFR signaling, we first used immunofluorescence microscopy to look for changes in cellular localization of Jab1 following treatment with EGF. We observed that EGF treatment was followed by increased translocation of Jab1 to the nucleus in both MDA-MB-231 and MDA-MB-468 breast cancer cell lines (Figure 1b). This effect is particularly evident in the merged images. Quantitative analysis of Jab1 nuclear expression confirmed that Jab1 levels were approximately two-fold higher following EGF treatment compared with untreated cells (Figure 1c). This difference was statistically significant in both cell lines tested (*P *< 0.01). These results were confirmed by immunoblots of extracts prepared from EGF-treated MDA-MB-231 cells, which showed increased nuclear Jab1 and a corresponding decrease in cytoplasmic Jab1 following EGF treatment (Figure 1d), as well as EGF dose-response experiments that showed increased nuclear Jab1 with increased EGF treatment concentration (Figure 1e). Similar observations were made in MDA-MB-468 cells (data not shown).

### Effect of EGF on Jab1 translocation is mediated through the ERK pathway

**Figure 2:**
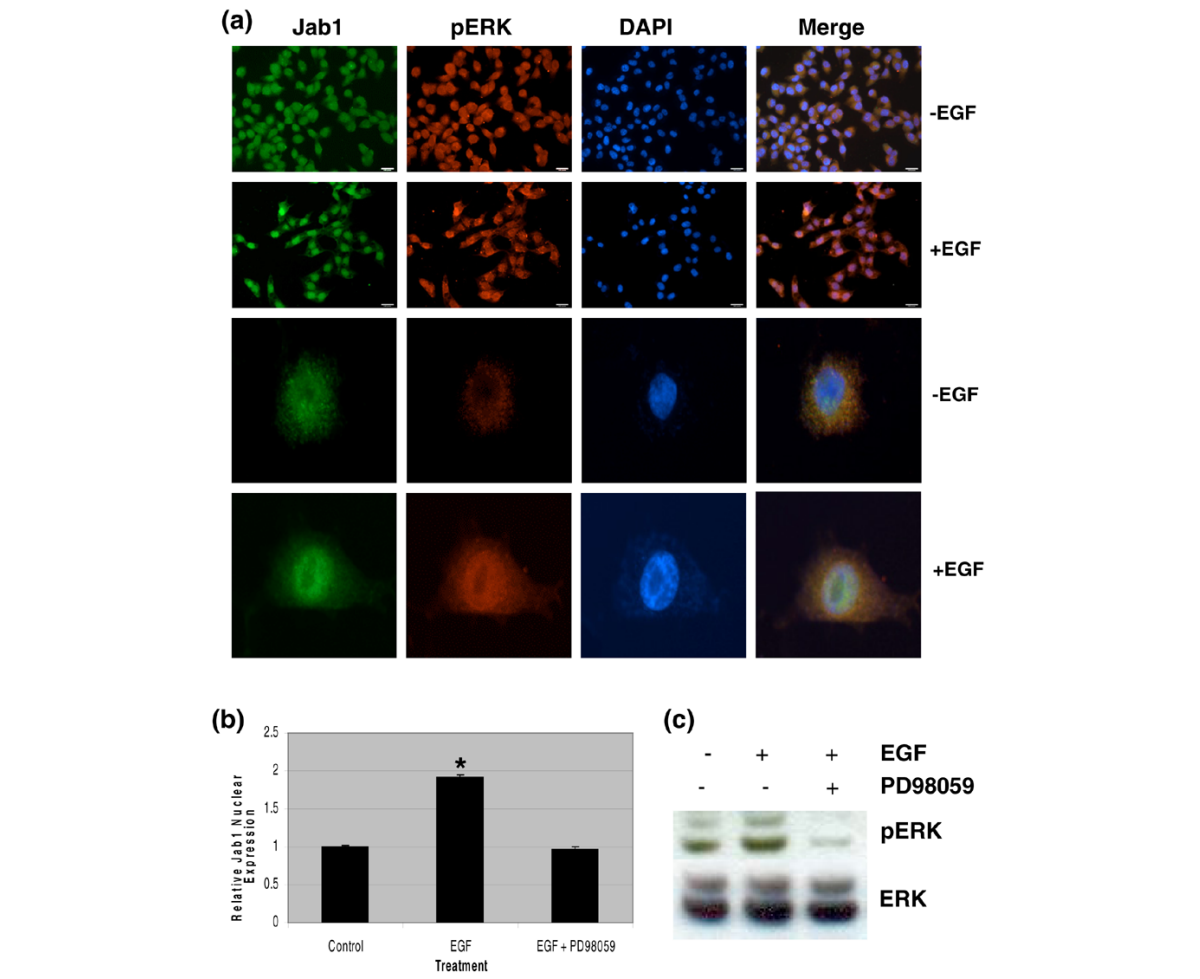
Epidermal growth factor (EGF) stimulation of Jab1 nuclear translocation is dependent on pERK and is associated with pERK in the nucleus in MDA-MB-231 cells. **(a) **Enhanced colocalization of Jab1 and pERK in the nucleus following EGF treatment. **(b) **Quantitative image analysis of nuclear Jab1 expression in cells treated with EGF and in cells treated with EGF and the ERK inhibitor, PD98059. Bars represent mean ± standard deviation. Statistical analysis was by *t *test (*P < 0.01). **(c) **Increased pERK expression following EGF treatment. The addition of ERK inhibitor, PD98059, eliminated this effect. DAPI, 4'-6-diamidino-2-phenylindole; ERK, extracellular signal-regulated kinase; Jab1, c-Jun activation domain-binding protein-1; pERK, phosphorylated extracellular signal-regulated kinase.

The effects of EGF are known to be mediated through the EGFR and by mitogen-activated protein (MAP) kinases [33]. We therefore examined whether the effect of EGF on Jab1 translocation is dependent on selective activation of the MAP kinases: p38, c-jun N-terminal kinase (JNK), and ERK. Experiments in our breast cancer cell lines showed that EGF treatment significantly increased phosphorylation of ERK as measured by immunofluorescence (Figure 2a). Minimal effects of EGF treatment were observed on phosphorylation of p38 and JNK (data not shown). We next looked at the localization of Jab1 and phosphorylated ERK (pERK). Double-immunostaining for these proteins showed that, following EGF treatment, there was an increase in both Jab1 and pERK and that these proteins were colocalized in the nucleus (Figure 2a, bottom row). ERK inhibitor, PD98059, was used in conjunction with EGF stimulation and was shown to effectively block increased nuclear Jab1 expression in MDA-MB-231 cells by both immunofluorescence (Figure 2b) and immunoblotting (Figure 2c). Similar observations were made in MDA-MB-468 cells (data not shown). These results indicate that EGF-induced Jab1 translocation can be mediated through the ERK signaling pathway.

### EGFR signaling regulates genes downstream of Jab1

**Figure 3:**
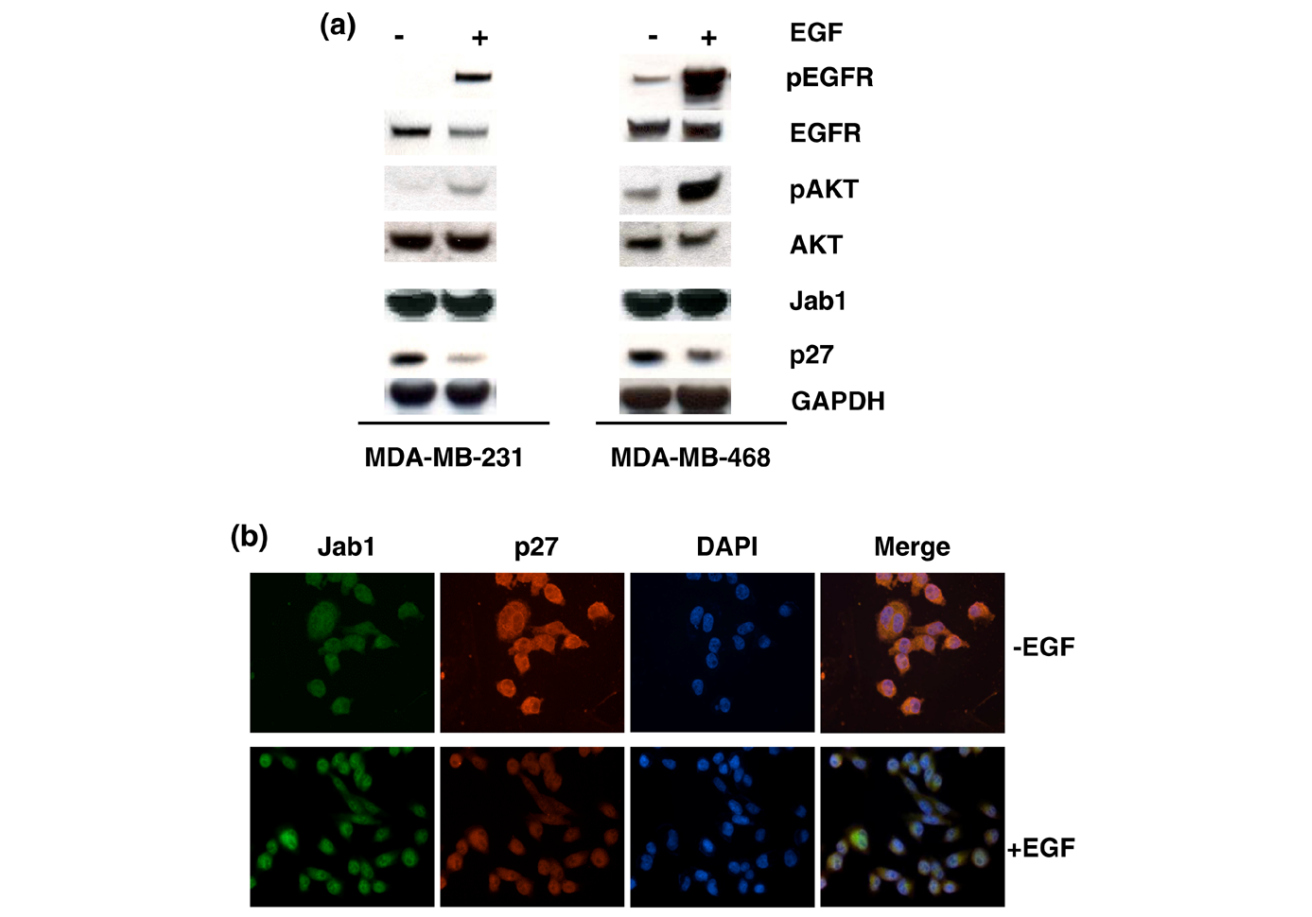
Epidermal growth factor (EGF) influences activity of Jab1 downstream genes. **(a) **Western blot analysis results show that upregulation of pEGFR is correlated with downregulation of p27 in both MDA-MB-231 and MDA-MB-468 cells following EGF treatment. Treatment with EGF was also correlated to an observed increase in pAKT. GAPDH was included as a loading control. **(b) **Immunofluorescence analysis confirmed that nuclear translocation of Jab1 was associated with downregulation of its downstream target, p27, following EGF treatment. DAPI, 4'-6-diamidino-2-phenylindole; EGFR, epidermal growth factor receptor; GAPDH, glyceraldehyde 3-phosphate dehydrogenase; Jab1, c-Jun activation domain-binding protein-1; pEGFR, phosphorylated epidermal growth factor receptor.

**Figure 4:**
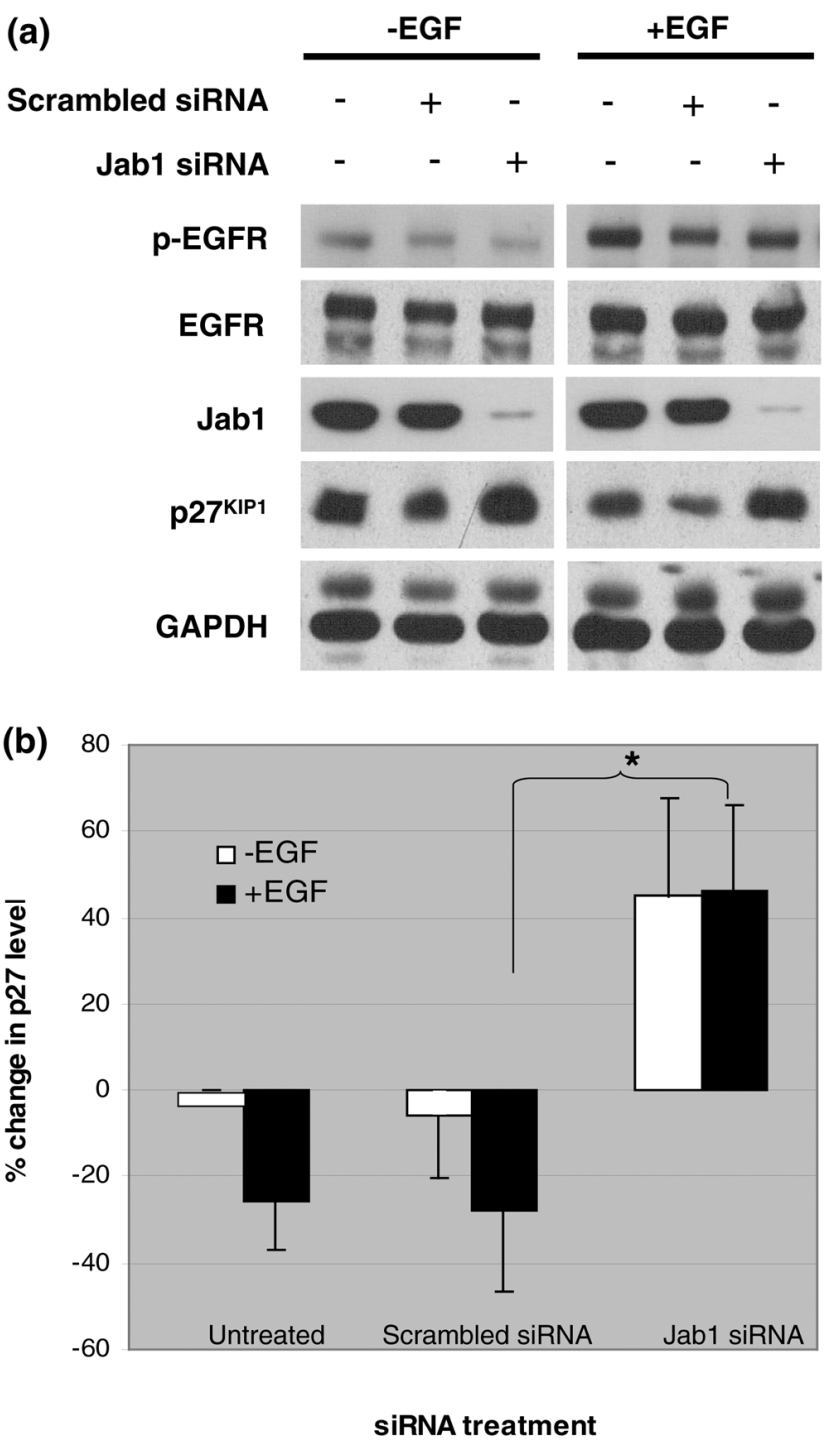
Effect of Jab1 short interfering RNA (siRNA) knockdown on p27 levels with and without epidermal growth factor (EGF) treatment in MDA-MB-231 cells. **(a) **Western blot analysis of cell lysates collected following the indicated treatments. Relative p27 band intensities were calculated by densitometry and normalized to the loading control (GAPDH). **(b) **The percentage change in p27 expression for EGF-treated cells relative to untreated controls. Data shown are representative of several independent experiments, demonstrating that knockdown of Jab1 abrogates EGF-induced downregulation of p27. Bars represent mean ± standard deviation. Statistical analysis was by *t *test (*P < 0.0001). All EGF treatments were at 50 ng/mL for 4 hours. EGFR, epidermal growth factor receptor; GAPDH, glyceraldehyde 3-phosphate dehydrogenase; Jab1, c-Jun activation domain-binding protein-1; pEGFR, phosphorylated epidermal growth factor receptor.

To investigate whether EGFR signaling has a functional effect on Jab1 activity, we performed immunoblotting and double-immunostaining for the Jab1 downstream target, p27. In both MDA-MB-231 and MDA-MB-468 cell lines, Western blot assay showed that EGF treatment and phosphorylation of EGFR resulted in a significant decrease in p27 expression (Figure 3a). Additional observed changes following EGF treatment included increased pAKT (Figure 3a). The inverse correlation between nuclear Jab1 and p27 expression was also observed in double-immunostaining for these proteins (Figure 3b). To confirm that Jab1 was necessary for the effect of EGF on p27, we performed Jab1 knockdown using an siRNA approach in MDA-MB-231 cells in conjunction with EGF treatment. In addition to re-confirming that cells treated with EGF have reduced p27 (*P *< 0.05), we found that Jab1 knockdown restored p27 to EGF-untreated levels compared with cells treated with EGF and control siRNA (*P *< 0.0001) (Figure 4a,b). In cells treated with Jab1 siRNA, EGF had no effect on p27 levels (*P *= 0.68). Taken together, these results indicate not only that EGFR signaling affects Jab1 translocation but that it may regulate targets downstream of Jab1 and that the effect of EGF on p27 levels is mediated by Jab1.

### Jab1 expression correlates with EGFR in breast tumors

**Figure 5:**
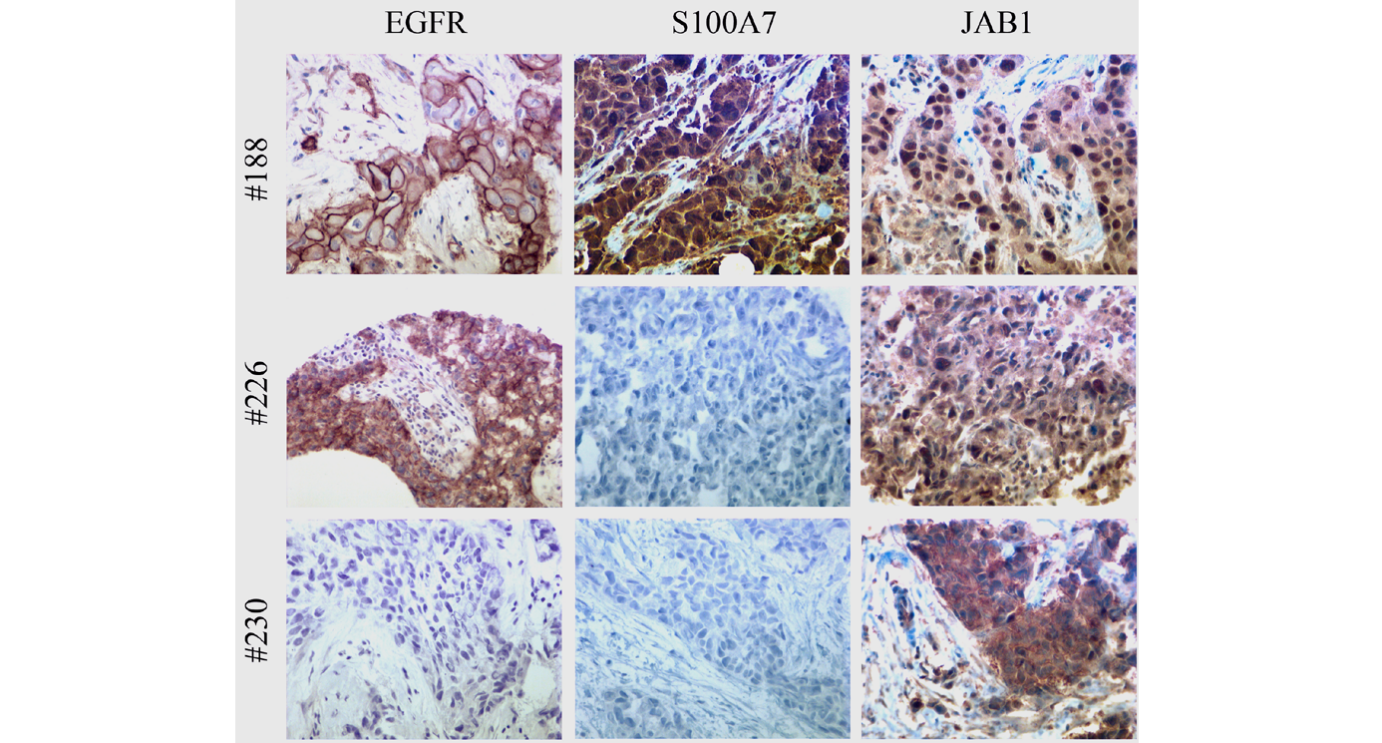
Jab1 nuclear expression correlates with increased EGFR and S100A7 in breast tumors. EGFR, S100A7, and Jab1 were detected by immunohistochemistry in breast tumors represented within a tissue microarray. Representative staining is shown for all three markers and in three separate tumors. Tumor #188 (top row) shows high nuclear Jab1 expression associated with high EGFR and S100A7 expression. Tumor #226 (middle row) shows intermediate nuclear and cytoplasmic Jab1 expression associated with moderate EGFR and absence of S100A7 expression. Tumor #230 (bottom row) shows low nuclear but high cytoplasmic Jab1 expression associated with the absence of EGFR or S100A7 expression. EGFR, epidermal growth factor receptor; Jab1, c-Jun activation domain-binding protein-1.

To further explore the relationship between EGFR and Jab1 expression *in vivo*, we examined the expression of these genes in a series of 424 invasive breast tumors using TMAs. The characteristics of the cohort are outlined in Table 1. The relationship between nuclear expression of Jab1 and the level of EGFR was assessed, together with the level of S100A7, because of the previously established strong relationship between S100A7 expression and Jab1. In analysis of the entire tumor cohort, high levels of Jab1, EGFR, and S100A7 were seen in 154/424 (36%), 42/424 (10%), 144/424 (34%) cases, respectively (Figure 5). Jab1 was not associated with prognostic factors or biomarkers, including grade, axillary nodal status, tumor size, ER, PR, EGFR, or S100A7, or with overall patient survival when examined in the entire cohort. In subgroup analysis of the ERα^+ ^subgroup, no significant associations were observed. However, in subgroup analysis of the ERα^- ^subgroup (n = 154), Jab1 levels were associated with axillary node-positive status (*P *= 0.019, *t *test) and higher levels of Jab1 nuclear expression were associated with both EGFR (*P *= 0.019, *t *test) and S100A7 (*P *= 0.036, *t *test) (Table 2). Notably, higher Jab1 levels were more strongly associated with combined EGFR^+^/S100A7^+ ^versus EGFR^-^/S100A7^- ^status within this subgroup (*P *= 0.004, *t *test).

**Table 1:** Clinicopathological features of the tumor cohort

Parameter		Number	Percentage
Grade^a^			
	Low	69	16%
	Intermediate	237	56%
	High	90	21%
	Unknown	28	7%
Size			
	≤2 cm	115	27%
	>2 cm	304	71%
	Unknown	5	2%
Nodal status			
	Negative	163	38%
	Positive	249	59%
	Unknown	12	3%
Estrogen receptor^b^			
	Negative	154	36%
	Positive	270	64%
Progesterone receptor^c^			
	Negative	189	45%
	Positive	235	55%

^a^Based on Nottingham system. ^b^Estrogen receptor-negative, <3 fmol/mg protein. ^c^Progesterone receptor-negative, <15 fmol/mg protein.

**Table 2:** Correlation between Jab1 expression and clinicopathological features of the estrogen receptor-alpha-negative^a ^subset

			Jab1		
Parameter		Number	Mean	(Standard deviation)	*P *value
Grade^b^					
	Low	12	91	(79)	NS
	Intermediate	70	61	(51)	
	High	72	49	(43)	
Size					
	≤2 cm	32	71	(63)	NS
	>2 cm	118	55	(50)	
Nodal status					
	Negative	55	43	(46)	0.019
	Positive	87	64	(55)	
Progesterone receptor^c^					
	Negative	120	59	(51)	NS
	Positive	34	53	(57)	
EGFR					
	Negative	123	53	(50)	0.019
	Positive	31	78	(57)	
S100A7					
	Negative	59	48	(47)	0.036
	Positive	95	64	(55)	

^a^Estrogen receptor-negative, <3 fmol/mg protein. ^b^Based on Nottingham system. ^c^Progesterone receptor-negative, <15 fmol/mg protein. EGFR, epidermal growth factor receptor; NS, not significant.

Outcome analysis of the ERα^- ^subgroup showed no significant association between survival and Jab1^+^, EGFR^+^, or S100A7^+ ^status when each marker was analyzed independently. Comparison of the subset of ERα^- ^tumors that were positive for all three markers, EGFR/S100A7/Jab1, showed that this phenotype was associated with worse outcome compared with EGFR/S100A7/Jab1^- ^tumors (n = 10 versus 13; *P *= 0.07).

## Discussion

ERα^-^, and in particular the 'triple-negative' subset of breast cancer lacking detectable ERα, PR, and Her2, has emerged as a challenge for systemic therapy now that successful targeted therapies have become available for the treatment of other phenotypic subgroups. Nevertheless, one prominent feature of the ERα^- ^subgroup is expression of the EGFR [34,35], raising the possibility that this receptor may offer a target for treatment of this subgroup. However, anti-EGFR therapies, alone or in combination with chemotherapy, have benefited only a small cohort of patients in the face of both *de novo *and acquired resistance to these therapies [36]. To circumvent this resistance, it will be important to understand more of the signaling pathways downstream of EGFR in ERα^- ^tumors [37]. Recent findings suggest that the Jab1 protein may be the central mediator in several of the biological circuits that promote tumor progression in breast cancer cells [37]. We have therefore set out to explore whether Jab1 is also involved in EGFR signaling. We have shown that EGFR activation in ERα^- ^breast cell lines is associated with Jab1 nuclear localization and that these changes relate to activation of both AKT and ERK pathways and modulation of Jab1 downstream genes. Furthermore, higher Jab1 nuclear expression was associated with EGFR^+ ^status in a cohort of ERα^- ^breast tumors, and this relationship was most significant in tumors that expressed both EGFR and S100A7 markers.

Jab1 is a multifunctional protein that has been shown to interact with several components of cell signaling pathways within *in vitro *yeast systems and human cell lines. These interactions usually are associated with translocation of Jab1 from the cytoplasm to the nucleus and result in either enhanced activity of transcription factors, including c-Jun [26,38], AP-1 [26,38], HIF-1α [39], steroid receptors, and cofactors [40], or the promotion of degradation of interacting proteins, including p27 [27,32], Smad4 [28], MIF1 [41], and p53 [42]. Although the physiological relevance of some of these interactions is mostly unknown (specifically, within the context of breast epithelial cells [43]), they are evidently complex. For example, in documenting that EGF can affect Jab1 localization in breast cells, we have confirmed previous findings that EGF affects a representative Jab1 downstream gene, p27, and that these effects correlate with alterations of PI3K (phosphatidylinositol-3-kinase)/AKT [44,45]. However, we also show here that changes in the ERK pathway may contribute to the effects of Jab1 in some breast cell lines. Interestingly, others recently have shown that Her2 (EGFR2) signaling can regulate Jab1 through the AKT/β-catenin pathway [23] and, in a subsequent study, that Her2 modulates p27 through Jab1 [23]. In contrast to our data and other interaction effects, these studies concluded that Her2-mediated Jab1 regulation occurs at the transcriptional level. Others have shown Her2 activation to be associated with relocalization to the cytoplasm rather than nuclear accumulation of Jab1 [46] and that activation of the Her2-ras-MAP kinase pathway can alter Jab1 and stimulate downregulation of p27 [47]. One potential explanation for these apparent incongruities relates to the different cell lines used in these studies [23].

Jab1 recently has been identified as a master regulator of a spectrum of genes (the 'wound response signature') that may promote tumor progression in breast cancer [24,25]. Jab1 also acts as an essential modulator of c-myc transcriptional activity, regulating c-myc protein ubiquitination and stability. Thus, Jab1 and c-myc together influence the expression of a subset of c-myc-regulated genes that comprise the 'wound response'. Jab1 and c-myc expression and upregulation of the wound response signature do not appear to be limited to specific phenotypic subgroups of breast tumors [48]. However, deregulation of c-myc is known to occur in ERα^- ^breast cell lines and to be associated with PR^- ^breast cancer and resistance to endocrine therapy [49,50]. We have previously identified Jab1 as a mediator of several intracellular and biological effects of S100A7, which itself may promote breast tumor progression [21,22]. Pathways downstream of S100A7 are also of interest because of this gene's strong association with the ERα^- ^phenotype in DCIS and, when expression persists, in invasive breast cancer [7,8,18,19].

These observations raise the question of whether Jab1 is a common factor in mediating cell signaling pathways that are important in ERα^- ^breast cancer. Our data presented here suggest that Jab1 may be regulated by the EGFR and S100A7 pathways in ERα^- ^breast cells. Notably, we [6] and (very recently) others [51] have shown that there may be crosstalk between S100A7 and EGFR and that S100A7 can regulate EGFR signaling. Jab1 expression in breast cancer has been explored previously by us [52] and others [23,29,53]. High nuclear Jab1 was associated with reduced p27 expression in all of these studies, in both DCIS [52] and invasive disease [29,53]. But no consistent association with any prognostic features, including ERα status, has emerged. However, there is some indication that increased Jab1 might be related to poor outcome [29]. Nevertheless, these studies were based on small [23] and/or highly selected case series (in terms of stage [52] and nodal status [29,53]). The present study has now extended these findings by assessing nuclear Jab1 expression in relation to prognostic features and markers in a large cohort of invasive breast tumors representative of the case distribution in a large tumor bank. We have confirmed that Jab1 is not strongly correlated with any prognostic features examined, except in subset analysis in which there was a positive association with nodal metastasis in the ERα^- ^subset. Despite the observation of a possible association between Jab1 and worse outcome in the ERα^- ^subset, this was not statistically significant, and the same was true for EGFR and S100A7. This difference from previous findings [6,29,54] may relate to the use of a TMA for the present study. While this format is optimal for examining coexpression of biomarkers within small defined tumor regions, it may not be optimal for outcome analyses of genes that are heterogeneously expressed within tumors. However, the aggregate results from this and other studies support the conclusion that nuclear Jab1 is only weakly related, if at all, to standard prognostic features and outcome as an independent factor.

This lack of clear association with complex phenotypic traits represented by prognostic factors such as tumor grade or with patient outcome is intriguing given the range of potentially important signaling pathways and proteins that Jab1 influences. On the other hand, it is perhaps not surprising given that these multiple factors may influence the equilibrium between nuclear and cytoplasmic Jab1 and its activity. It has also been shown that p53 and c-Jun can compete for Jab1 [47]. These and other interacting proteins might influence its collaborative role with c-myc as a regulator of the wound response. Jab1 can also exist in several different protein complexes within both cellular compartments in breast cells, further complicating analysis and deductions based only on protein localization [25]. It will be interesting to examine Jab1 in relation to c-myc and Jab1 protein complex status in future outcome analyses.

## Conclusion

Jab1 lies at the intersection of several signaling pathways that are believed to be important in breast cancer cells and may be a decisive influence on the outcome of specific pathway alterations and their cumulative effects on progression. Our results implicating Jab1 in the EGFR pathway, in addition to its role in the S100A7 pathway, suggest that Jab1 may be particularly important in the ERα^- ^breast cancer cell and provide insight into the application of new therapeutic strategies (for example, proteasome inhibitors) directed to this important and difficult-to-treat subset of breast cancer.

## Abbreviations

AP-1 = activation protein-1; DCIS = ductal carcinoma *in situ*; EDTA = ethylene diamine tetraacetic acid; EGF = epidermal growth factor; EGFR = epidermal growth factor receptor; EGTA = ethylene glycol tetraacetic acid; ER = estrogen receptor; ERK = extracellular signal-regulated kinase; GAPDH = glyceraldehyde 3-phosphate dehydrogenase; HEPES = 4-(2-hydroxyethyl)-1-piperazineethanesulfonic acid; Her2 = epidermal growth factor receptor 2; IHC = immunohistochemistry; Jab1 = c-Jun activation domain-binding protein-1; JNK = c-jun N-terminal kinase; LBA = ligand-binding assay; MAP = mitogen-activated protein; NF-κB = nuclear factor-kappa B; PBS = phosphate-buffered saline; PBST = phosphate-buffered saline + 0.05% Tween-20; pERK = phosphorylated extracellular signal-regulated kinase; PR = progesterone receptor; siRNA = short interfering RNA; TMA = tissue microarray.

## Competing interests

The authors declare that they have no competing interests.

## Authors' contributions

PHW supervised all aspects of this study and contributed to the manuscript preparation. JW participated in the overall experimental design and data interpretation. ROB participated in the manuscript preparation and data interpretation. NRW performed all of the knockdown experiments and participated in the data interpretation. MO performed most of the cell culture and immunofluorescence assays. JEC performed most of the Western blot analyses. All authors read and approved the final manuscript.
